# ﻿A taxonomic revision of *Keraunea*, including three new species and its phylogenetic realignment with Ehretiaceae (Boraginales)

**DOI:** 10.3897/phytokeys.219.101779

**Published:** 2023-02-20

**Authors:** Peter W. Moonlight, Domingos Benício Oliveira Silva Cardoso

**Affiliations:** 1 Botany Department, School of Natural Sciences, Trinity College Dublin, Dublin 2, Ireland Trinity College Dublin Dublin Ireland; 2 Instituto de Pesquisas Jardim Botânico do Rio de Janeiro, Rio de Janeiro, Brazil Instituto de Pesquisas Jardim Botânico do Rio de Janeiro Rio de Janeiro Brazil; 3 Universidade Federal da Bahia, Salvador, Bahia, Brazil Universidade Federal da Bahia Salvador Brazil

**Keywords:** Boraginales, Brazil, Caatinga, Ehretiaceae, *Keraunea*, Mata Atlantica

## Abstract

*Keraunea* is an enigmatic genus of lianescent shrubs endemic to Brazil and found within the Caatinga and Mata Atlântica phytogeographic regions. When first published, *Keraunea* was included in the Convolvulaceae and there has been considerable recent disagreement about its true family placement on the Angiosperm tree of life. Based on further assessment of morphology and a new comprehensively-sampled combined phylogenetic analysis of nuclear and plastid genes from recently published DNA sequence data, we settle the position of the genus within the Ehretiaceae as sister to the Australian genus *Halgania* Gaudich. and provide an expanded family description. We recognize five species within *Keraunea*, three of them newly described here: *K.brasiliensis* Cheek & Simão-Bianchini, *K.bullata* Moonlight & D.B.O.S.Cardoso, **sp. nov.**, *K.capixaba* Lombardi, *K.confusa* Moonlight & D.B.O.S.Cardoso, **sp. nov.** and *K.velutina* Moonlight & D.B.O.S.Cardoso, **sp. nov.** We also provide a full taxonomic revision of the genus, including a key, descriptions, map of geographical distribution and provisional IUCN threat assessments for all species.

## ﻿Introduction

In spite of its short taxonomic history, the small Brazilian genus *Keraunea* Cheek & Simão-Bianchini (Cheek and Simão-Bianchini 2013) has caused considerable taxonomic confusion. In 2013, three specimens of lianescent shrubs with a highly unusual inflorescence structure were published as a new genus and species: *Kerauneabrasiliensis* Cheek & Simão-Bianchini (Cheek and Simão-Bianchini 2013). The authors ”confidently” placed the genus within the Convolvulaceae because its fruits (with the pedicels adnate to a large leaf-like bracteole) resembled the African lianas of the genus *Neuropeltis* Wall. Additional flowering, fruiting and vegetative characters were used to support this family placement (Cheek and Simão-Bianchini 2013). In 2014, a second species of *Keraunea* was described, *K.capixaba* Lombardi (Lombardi 2014). This was based upon four specimens, all collected after 2012 in Jaguaré and Sooretama municipalities, Espírito Santo, Brazil.

The first molecular phylogenetic study that sampled *Keraunea* was published in 2022 by authors working on the Convolvulaceae of the Americas (Muñoz-Rodríguez et al. 2022). The authors produced DNA sequence data for an isotype (*Passos et al. 5263* [K]) and paratype (*Lombardi & Salino 1819* [K]) of *K.brasiliensis* and a paratype of *K.capixaba* (*Siqueira 893* [K]). Rapid phylogenetic inference was carried out with IQ-Tree (Nguyen et al. 2015), which conclusively demonstrated that all samples fell outside of the Convolvulaceae. More surprisingly, the isotype of *K.brasiliensis* was resolved within the Malpighiaceae while the paratype of *K.brasiliensis* and the paratype of *K.capixaba* was resolved within the Ehretiaceae. It is worth noting, however, that the primary motivation for this study was demonstrating that *Keraunea* does not belong within the Convolvulaceae, and the authors suggested further comprehensive studies were required to correctly place *Keraunea* in the right family (Muñoz-Rodríguez et al. 2022). The placements within the Malpighiaceae and Ehretiaceae were well supported but the authors did not formalise the taxonomic changes because of their conflicting results and the sparse phylogenetic sampling within the Ehretiaceae. The placement with *Keraunea* nested in Malpighiaceae has more recently shown to be erroneous, possibly caused by Muñoz-Rodríguez et al. (2022) extracting DNA from an errant Malpighiaceae leaf, likely *Masganiacordifolia* (A.Juss.) Griseb., found within the capsule of the Kew specimen of *Passos et al. 5236* (de Almeida et al. 2023). While we note that Muñoz-Rodríguez et al. (2022) did re-extract and re-sequence the specimen *Passos et al. 5236* (K), it is unclear whether this was from the same leaf sample.

During general collecting in the Caatinga of southwestern Bahia and northern Minas Gerais, the second author of this paper collected several specimens of two closely related species but was unable to determine them to family with confidence. These were identified subsequently as *Keraunea* with reference to the specimens in HUEFS but, like the authors of Muñoz-Rodríguez et al. (2022), we were unconvinced by placement in the Convolvulaceae. Furthermore, one of our yet undescribed species was a close match for the type of *K.brasiliensis* (*Passos et al. 5263*) but the second was a close match for a paratype of that species (*Lombardi & Salino 1819*). This led us to conclude that the original generic description and illustration of *K.brasiliensis* (Cheek and Simão-Bianchini 2013) were based upon two separate species, both of which were distinct from *K.capixaba*. We then conducted a thorough survey and revision of the material held in several Brazilian herbaria (ALCB, HUEFS, RB) and online during which we were able to recognise five species of *Keraunea*.

It came to our attention after the submission of our article that a preprint had been filed with bioRxiv, also placing *Keraunea* within the Ehretiaceae and providing a taxonomic revision of the group (Cheek et al. 2023). While not effectively published, this article includes the description of “*K.lombardiana* Cheek”, whose type specimen falls within our concept of *K.confusa* Moonlight & D.B.O.S.Cardoso but also includes material from our concept of *K.bullata* Moonlight & D.B.O.S.Cardoso (*Rose & Russell 19979*, *Mori et al. 11534*). The article also included “*Keraunea* sp. A” whose concept matches our *K.velutina* Moonlight & D.B.O.S.Cardoso, and “*Keraunea* sp. B” whose concept falls clearly within our concept of *K.capixaba*.

In this article we assemble a more comprehensive and taxonomically vetted molecular dataset based on four genes to settle the family-level placement and generic relationships of *Keraunea*. We also provide an expanded description of the Ehretiaceae and the genus *Keraunea*. We further provide a full taxonomic revision of the genus, including a key to all five species, and the publication of three undescribed species. For each species, we include a description, provisional IUCN Red List assessment, and identification notes.

## ﻿Methods

### ﻿Taxonomic treatment

We performed a thorough search of specimens available in several Brazilian herbaria (all herbarium acronyms follow *Index herbariorum*, Thiers 2016) supplemented with searches of the online databases CRIA Species Link (http://splink.cria.org.br/, February 2023) and Flora do Brasil (http://floradobrasil.jbrj.gov.br/reflora/herbarioVirtual/, February 2023). We searched for all Brazilian data of *Keraunea* and Ehretiaceae as well as undetermined material in the families Boraginaceae, Dichapetalaceae, and Nyctaginaceae (all families into which *Keraunea* specimens had previously been thought to belong). All descriptions are based upon direct measurements of herbarium specimens and our own field collections, with some macro-morphological characters measured from images of herbarium specimens using the ruler tool in Adobe Photoshop CS5 v.12.0 ×64.

### ﻿Phylogenetic analyses

Previously published molecular phylogenetic analyses have suggested that the genus *Keraunea* is a member of the Boraginales and more closely related to the Ehretiaceae than to Malpighiacae (Muñoz-Rodríguez et al. 2022; de Almeida et al. 2023). As such, we assembled a new, more comprehensively-sampled combined molecular dataset of the plastid regions *matK*, *rbcL* and *trnL-F* and the nuclear ribosomal internal transcribed spacer (ITS), with focus on members of the Ehretiaceae. The molecular data were downloaded from the GenBank sequence repository (http://www.ncbi.nlm.nih.gov/Genbank), including one published *Keraunea* sample (Muñoz-Rodríguez et al. 2022). We supplemented these data with eight samples representing families in the Boraginales*sensu*Luebert et al. (2016). All sequences were retrieved with a custom R script using functions from ape package (Paradis and Schliep 2019). Details of all sequenced samples and their GenBank accession numbers are available in Table 1.

**Table 1.** Details of all sequenced samples used in the phylogenetic analysis (Fig. 1), including species names, voucher information and GenBank accession numbers.

Family	Species	Voucher	*trnL-F*	*rbcL*	*matK*	ITS
Boraginaceae	*Pentaglottissempervirens* (L.) L.H.Bailey	*Weigend 9065* (B)	KF158194	KF158095	NA	NA
Codonaceae	*Codonroyenii* L.	*Greuter 21551* (B)	KC542572	KF158090	NA	NA
Cordiaceae	*Coldeniaprocumbens* L.	*Jongkind 1973* (MO)	KF158207	KF158128	DQ197227	DQ197284
Heliotropiaceae	*Heliotropiumstenophyllum* Hook. & Arn.	*Luebert* 1990 (SGO)	EF688847	KF158148	NA	EF688899
Hydrophyllaceae	*Eriodictyontrichocalyx* A.Heller	*Fraga 2209*	KX929271	KX929185	NA	NA
Lennoaceae	*Pholismaarenarium* Nutt. ex Hook.	*Hilger 1992/62* (B)	KF158216	KF158155	DQ197226	DQ197283
Wellstediaceae	*Wellstediasomalensis* Thulin & A.Johanss.	*Thulin 10084* (UPS)	KF158198	KF158105	NA	NA
Ehretiaceae	*Bourreriaapetala* (J.S.Mill.) J.S.Mill. & Gottschling	*Jongkind 3280* (MO)	NA	NA	NA	KF673262
Ehretiaceae	*Bourreriabosseri* (J.S.Mill.) J.S.Mill. & Gottschling	*Miller 6182* (MO)	NA	NA	NA	KF673265
Ehretiaceae	*Bourreriacroatii* (J.S.Mill.) J.S.Mill. & Gottschling	*Miller 4514* (MO)	NA	NA	NA	KF673263
Ehretiaceae	*Bourreriaexsucca* Jacq.	*Ramirez 2744* (MO)	KF673291	NA	NA	KF673264
Ehretiaceae	*Bourreriahavanensis* (Willd. ex Roem. & Schult.) Miers	*Abbott 23888* (FLAS)	NA	KJ773317	KJ772588	NA
Ehretiaceae	*Bourrerialittoralis* Urb.	*BioBot00944*	NA	JQ590874	JQ587063	NA
Ehretiaceae	*Bourreriamoaensis* Britton	*Beurton s.n.* (B)	KF158205	KF158116	NA	KF673250
Ehretiaceae	*Bourreriamollis* Standl.	*Wallnöfer 9717* (MO)	KF673283	NA	NA	AF385780
Ehretiaceae	*Bourreriapetiolaris* (Lam.) Thulin	*Hilger s.n.* (B)	NA	NA	NA	KF673248
Ehretiaceae	*Bourreriapulchra* Millsp.	*Sima 2101* (F)	KF673288	NA	NA	KF673259
Ehretiaceae	*Bourreriaquirosii* Standl.	*Gomez 18666* (MO)	KF673290	NA	NA	KF673261
Ehretiaceae	*Bourreriaspathulata* (Miers) Hemsl.	*Cedillo 1415* (MO)	KF673289	NA	NA	KF673260
Ehretiaceae	*Bourreriasucculenta* Jacq.	*Olmstead 96/114* (WTU)	NA	NA	DQ197229	DQ197285
Ehretiaceae	*Ehretiaacuminata* R.Br.	*Anonymous B227*	EU599919	EU599831	EU599655	NA
Ehretiaceae	*Ehretiaamoena* Klotzsch	*Maurin 1123*	NA	JF265404	JF270754	NA
Ehretiaceae	*Ehretiaanacua* (Terán & Berl.) I.M.Johnst.	*Cultivated HB Adelaide*	EU600009	EU599833	EU599657	AF385796
Ehretiaceae	*Ehretiaaquatica* (Lour.) Gottschling & Hilger	*Jongkind 2517* (MO)	EU600011	EU599835	EU599659	AF385791
Ehretiaceae	*Ehretiaaspera* Willd.	*Rechinger 29501* (B)	KF673273	NA	NA	AF385787
Ehretiaceae	*Ehretiabakeri* Britten	*Chen 28823*	NA	ON950787	ON981999	NA
Ehretiaceae	*Ehretiacoerulea* Gürke	*Anonymous 1969* (B)	KF673274	NA	NA	KF673249
Ehretiaceae	*Ehretiacymosa* Thonn.	*De Wilde 4230* (B)	EU600012	EU599836	EU599660	AF385790
Ehretiaceae	*Ehretiadicksonii* Hance	*TNM S159846*	NA	MF623374	MF651973	NA
Ehretiaceae	*Ehretiaglandulosissima* Verdc.	*Chen 29046*	NA	ON950788	ON982000	NA
Ehretiaceae	*Ehretiagrahamii* Randell	*Anonymous 300101051*	NA	KU564793	KU564569	NA
Ehretiaceae	*Ehretialatifolia* DC.	*Roitman 18x96* (B)	KF673282	NA	NA	AF385797
Ehretiaceae	*Ehretialongiflora* Champ. ex Benth.	*King s.n.* (B)	EU600010	EU599834	EU599658	AY331400
Ehretiaceae	*Ehretiamacrophylla* Wall.	*XA010*	NA	MH657238	MH658814	MH712731
Ehretiaceae	*Ehretiamicrocalyx* Vaupel	*Crosby 1027*	KX929266	KX929180	NA	NA
Ehretiaceae	*Ehretiamicrophylla* Lam.	*Cultivated HB Singapore*	KF158204	KF158114	NA	AF469166
Ehretiaceae	*Ehretiaobtusifolia* Hochst. ex. A.DC.	*Ehrobt 419*	NA	NA	NA	AY331401
Ehretiaceae	*Ehretiaphilippinensis* A.DC	*P2300*	NA	LC604229	LC604260	NA
Ehretiaceae	*Ehretiaresinosa* Hance	*TNM S159880*	NA	MF623351	MF651953	NA
Ehretiaceae	*Ehretiarigida* (Thunb.) Druce	*Schwerdtfeger s.n.* (B)	KF673278	NA	NA	AF385788
Ehretiaceae	*Ehretiasaligna* R.Br.	*Walter s.n.* (B)	KF673272	NA	NA	AF385786
Ehretiaceae	*Ehretiatinifolia* L.	*Anonymous 439*	HQ286270	KF158115	NA	AF385793
Ehretiaceae	*Ehretiawallichiana* Hook.f. & Thomson ex C.B.Clarke	*Ehrwal 1362*	NA	NA	NA	AY331402
Ehretiaceae	*Halganiaanagalloides* Engl.	*Strid 21757* (B)	MF423321	NA	NA	MF423308
Ehretiaceae	Halagnia *andromedifolia* Behr. & F.Muell. ex F.Muell.	*Strid 21146* (B)	KF673280	NA	NA	AF402584
Ehretiaceae	*Halganiabebrana* Oldfield & F.Muell.	*Lewis s.n.* (B)	MF423323	NA	NA	MF423312
Ehretiaceae	*Halganiabrachyrhyncha* P.G.Wilson	*Streimann 820* (A)	NA	NA	NA	MF423313
Ehretiaceae	*Halganiacyanea* Lindl.	*Greuter 18801* (B)	KF158209	KF158130	NA	KF673254
Ehretiaceae	*Halganiaglabra* J.M.Black	*Broadbent 2042* (A)	NA	NA	NA	MF423307
Ehretiaceae	*Halganiaintegerrima* Endl.	*Strid 21282* (MO)	MF423324	NA	NA	MF423315
Ehretiaceae	*Halganiaandromedifolia* Behr. & F.Muell. ex F.Muell.	*Eichler 19328* (B)	NA	NA	NA	KF673255
Ehretiaceae	*Halganiasolanacea* F.Muell.	*Mitchell 1090* (B)	NA	NA	NA	MF423311
Ehretiaceae	*Lepidocordiapunctata* Ducke	*Steyermark 88509* (NY)	KF673287	NA	NA	KF673257
Ehretiaceae	*Lepidocordiawilliamsii* (I.M.Johnst.) J.S.Mill.	*Nee 27982* (M)	NA	NA	NA	KM893038
Ehretiaceae	*Rochefortiaacanthophora* (DC.) Griseb.	*Liogier 23240* (NY)	NA	NA	NA	KM893039
Ehretiaceae	*Rochefortiabarloventensis* Irimia & Gottschling	*Liogier 32280* (MO)	NA	NA	NA	KM893065
Ehretiaceae	*Rochefortiacubensis* Britton & P.Wilson	*Maxon 1720* (US)	NA	NA	NA	KM893049
Ehretiaceae	*Rochefortiacuneata* Sw.	*Correll 51418* (F)	NA	NA	NA	KM893048
Ehretiaceae	*Rochefortiaoblongata* Urb. & Ekman	*Ekman 9224* (G)	NA	NA	NA	KM893061
Ehretiaceae	*Rochefortiaspinosa* (Jacq.) Urb.	*Hilger 99/20* (B)	KF673276	NA	NA	KF673251
Ehretiaceae	*Rochefortiastellata* Britton & P.Wilson	*Areces 30714* (JE)	NA	NA	NA	KM893070
Ehretiaceae	*Tiquiliacanescens* (DC.) A.T.Richardson	*Moore 239* (TEX)	NA	NA	DQ197230	DQ197312
Ehretiaceae	*Tiquiliaconspicua* (I.M.Johnst.) A.T.Richardson	*Moore 294* (TEX)	NA	NA	DQ197250	DQ197586
Ehretiaceae	*Tiquiliacuspidata* (I.M.Johnst.) A.T.Richardson	*Moore 223* (TEX)	NA	NA	DQ197247	DQ197540
Ehretiaceae	*Tiquiliadarwinii* (Hook.f.) A.T.Richardson	*Tye 573* (CDS)	NA	NA	DQ197248	DQ197541
Ehretiaceae	*Tiquiliaelongata* (Rusby) A.T.Richardson	*Moore 289* (TEX)	NA	NA	DQ197251	DQ197588
Ehretiaceae	*Tiquiliagossypina* (Wooton & Standl.) A.T.Richardson	*Moore 134* (TEX)	NA	NA	DQ197233	DQ197337
Ehretiaceae	*Tiquiliagreggii* (Torr. & A.Gray) A.T.Richardson	*Moore 133* (TEX)	NA	NA	DQ197231	DQ197325
Ehretiaceae	*Tiquiliahispidissima* (Torr. & A.Gray) A.T.Richardson	*Moore 131* (TEX)	NA	NA	DQ197240	DQ197423
Ehretiaceae	*Tiquilialatior* (I.M.Johnst.) A.T.Richardson	*Moore 216* (TEX)	NA	NA	DQ197244	DQ197538
Ehretiaceae	*Tiquiliamexicana* (S. Watson) A.T.Richardson	*Moore 245* (TEX)	NA	NA	DQ197236	DQ197372
Ehretiaceae	*Tiquilianuttallii* (Benth. ex Hook.) A.T.Richardson	*Moore 218* (TEX)	NA	NA	DQ197254	DQ197579
Ehretiaceae	*Tiquiliapalmeri* (A.Gray) A.T.Richardson	*Moore 197* (TEX)	NA	NA	DQ197252	DQ197581
Ehretiaceae	*Tiquiliaparonychioides* (Phil.) A.T.Richardson	*Moore 300* (TEX)	NA	NA	DQ197249	DQ197564
Ehretiaceae	*Tiquiliaplicata* (Torr.) A.T.Richardson	*Moore 196* (TEX)	NA	NA	DQ197246	DQ197570
Ehretiaceae	*Tiquiliapurpusii* (Brandegee) A.T.Richardson	*Moore 109* (TEX)	NA	NA	DQ197245	DQ197409
Ehretiaceae	*Tiquiliatuberculata* A.T.Richardson	*Moore 98* (TEX)	NA	NA	DQ197239	DQ197407
Ehretiaceae	*Tiquiliaturneri* A.T.Richardson	*Moore 89* (TEX)	NA	NA	DQ197237	DQ197398
Ehretiaceae	*Kerauneaconfusa* Moonlight & D.B.O.S.Cardoso	*Lombardi 1819* (BHCB)	NA	NA	submitted	OP034981

The individual DNA matrices were subjected to automatic multiple alignments in AliView v.1.26 (Larsson 2014), using the Muscle algorithm, but with subsequent manual editing to improve homology in more variable sites. Individual DNA alignments were combined into a concatenated dataset using the R package catGenes (https://github.com/DBOSlab/catGenes; Cardoso et al. 2020). The custom R script uses catGenes functions to automatically build the concatenated dataset by maximising the inclusion of taxa that are incomplete or with missing data (Wiens 2003, 2006).

A maximum likelihood (ML) phylogeny was inferred using RAxML (Stamatakis 2006) using the graphical user interface raxmlGUI v.2.0 (Edler et al. 2020), with the following settings: best-scoring ML tree starting with a random seed and utilizing the GTR + GAMMA nucleotide substitution model, with the gamma distribution and invariants sites estimated during the run; and 500 rapid bootstrap (BS) replicates to determine branch support. The phylogeny was rooted based on a clade including the Wellstediaceae, Boraginaceae*s.str.* and Codonaceae following Luebert et al. (2017).

## ﻿Results

### ﻿Phylogenetic analyses

**Figure 1.**
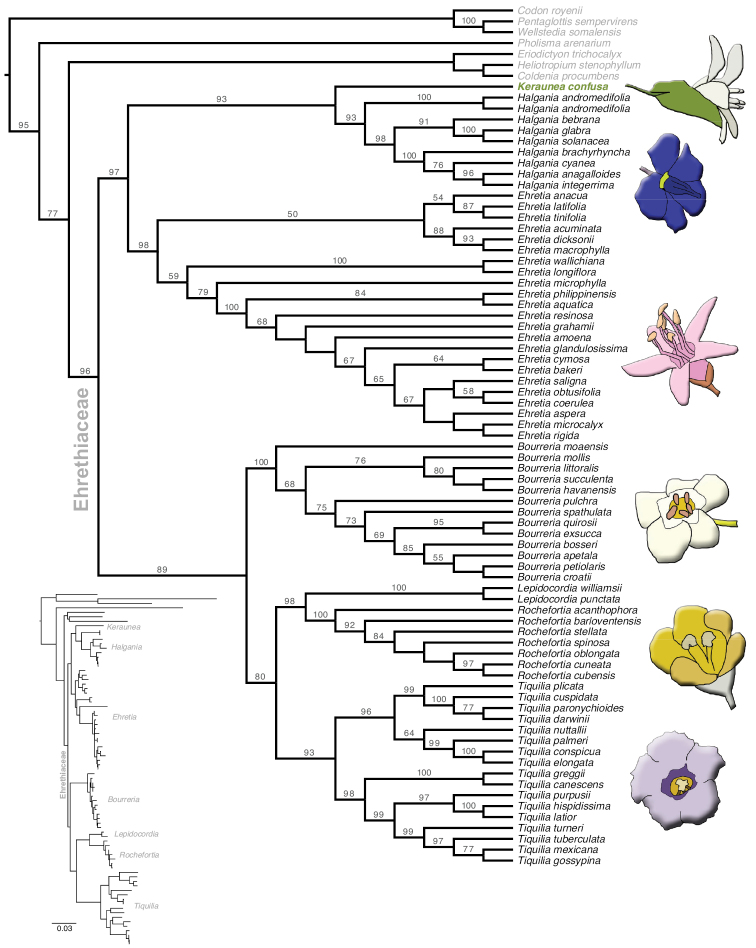
RAxML bootstrap tree based on nuclear ribosomal (ITS) and plastid (*matK*, *rbcL*, *trnL-F*) DNA sequence data resolving *Keraunea* within the Ehretiaceae. Node labels indicate nodes with bootstrap support values above 50. Inset phylogram (bottom left) indicates branch lengths. Outgroup taxa from representatives of other Boraginales families are in light grey. Illustrations are (top to bottom): *Keraunea*, *Halgania*, *Ehretia*, *Bourreria*, *Rochefortia* and *Tiquilia*.

Our four-gene ML phylogenetic analysis resolve the genus *Keraunea* as nested within the Ehretiaceae sister to the Australian endemic genus *Halgania* Gaudich. (Fig. 1). This placement is well supported (BS: 94) and consistent with previous analyses placing samples of the genus as nested within the Ehretiaceae (Muñoz-Rodríguez et al. 2022; Cheek et al. 2023). The topology within the Ehretiaceae is largely congruent with that found in previous analyses (e.g. Luebert et al. 2017) and the monophyly of the Ehretiaceae, including *Keraunea*, is well supported (BS: 97). The Ehretiaceae is resolved as sister to the Lennoaceae (BS: 96).

## ﻿Discussion

The phylogenetic realignment of the genus *Keraunea* as nested within the Ehretiaceae requires a slightly emended description of the family. *Keraunea* represents the first lianescent member of a family that otherwise includes trees, shrubs and perennial herbs (Luebert et al. 2016). The inflorescence structure of *Keraunea* is also unique not just within the Ehretiaceae but in the Boraginales as a whole. *Keraunea* does, however, present the only character believed to hold together the Ehretiaceae, bifid styles with two stigmatic branches (Luebert et al. 2016). We provide an emended family description below (see Taxonomic treatment) and a discussion of the unique inflorescence of *Keraunea*.

The placement of *Keraunea* as sister to the Australian endemic genus *Halgania* presents a biogeographical conundrum but is ecologically and morphologically coherent. *Halgania* is a dry adapted group (Holstein and Gottschling 2017), and *Keraunea* is primarily found in dry forest within the Caatinga phytogeographic domain or in and around rock outcrops in the Mata Atlântica phytogeographic domain. Insights from recent biogeographical studies with dated molecular phylogenies of transcontinentally disjunct plant clades have suggested that long distance dispersal and phylogenetic niche conservatism may explain why closely related lineages are ecologically confined to the same dry forest biome no matter how much geographic distance separates them (Gagnon et al. 2019; Ringelberg et al. 2020). An assessment of the diversification history of Ehretiaceae in the context of biome conservatism seems an important issue to explore in the future to better understand the evolution of the interesting geographical disjunction, yet similar ecologies of *Keraunea* and *Halgania*.

The flowers of *Halgania* are buzz-pollinated “solanum-type” flowers (Holstein and Gottschling 2018) that at first glance seem highly distinct from those of *Keraunea* (Fig. 1). The androecium of the two groups is very similar, with both genera having five stamens with short filaments fused at the base to the corolla tube, long anthers and conspicuous, extended connectives. The two genera differ in that the anthers of *Halgania* dehisce by opening into a small chamber formed by protrusions of the connective and appear to be poricidally dehiscent while those of *Keraunea* dehisce via longitudinal slits along the entire length of the anther, and in *Halgania* the anthers are held together in a cone by long, interlaced trichomes while in *Keraunea* they are held apart and are glabrous (Holstein and Gottschling 2018). Further, the fruits of the two genera are very similar. Cheek and Simão-Bianchini (2013) dissected a fruit of *K.brasiliensis* and characterised it as bilocular with four ovules, though two of those may be aborted. We characterise this fruit as a drupe, and bilocular, drupaceous fruits with four ovules are the most common character state in the Ehretiaceae (Luebert et al. 2016) and one shared with *Halgania* (Holstein and Gottschling 2018).

## ﻿Taxonomic treatment

We recognise five species of *Keraunea*, which are all endemic to Brazil, including three newly described species.

### ﻿ Ehretiaceae

Taxon classificationPlantaeBoraginalesEhretiaceae

Mart., Nov. Gen. Sp. Pl. 2: 136, 138. 1827 nom. cons.

F2F096E9-86A4-50F9-8431-06C6C6B21042

#### Type genus.

*Ehretia* P.Browne.

#### Description.

[differences from Luebert et al. (2016) in bold] Trees, shrubs, **lianas**, perennial herbs, rarely with thorns (*Rochefortia* Sw.); indumentum variable, hirsute to glabrescent. Leaves alternate, entire, petiolate; lamina variable in shape, strongly dissected in the halophytic *Cortesia* Cav. Inflorescences terminal or axillary thyrses, sometimes congested, **or few-flowered corymbs (*Keraunea*)**. Flowers pentamerous, cosexual or unisexual and dioecious in *Lepidocordia* Ducke and *Rochefortia*, **sometimes inserted on the centre of an accrescent bracteole (*Keraunea*)**; calyx lobes united in a tube or distinct nearly to the base, tubular to campanulate; aestivation imbricate (mostly quincuncial); corolla sympetalous, generally tubular with spreading lobes, rotate, or campanulate to urceolate, white, red or blue (*Halgania*, some species of *Bourreria* P.Browne); stamens 5, the filaments generally adnate to the corolla tube at least at the base, sometimes puberulent at the point of insertion, the anthers usually exerted; gynoecium bicarpellate, the ovary uni- to tetralocular from secondary subdivision, style terminal, the stigma clavate to capitate with 1(2) branches; nectar disc usually present at base of the ovary. Fruits drupaceous, often drying and separating into two two-seeded pyrenes, or 4 1-seeded pyrenes or schizocarps, or 4 nutlets.

#### Distribution.

Ehretiaceae is a broadly distributed family found throughout tropical and subtropical Asia, Australia, sub-Saharan Africa. In the Americas, its distribution encompasses the eastern United States, Florida, Central America, the Caribbean, the Guyana shield and the Andes. In Brazil, the Ehretiaceae was previously only known from the single species *Lepidocordiapunctata* Ducke (Stapf 2023), found in lowland Amazonian forests in Pará and Roraima states. Our treatment therefore represents new records of the family from the Caatinga and Mata Atlântica phytogeographic regions in Brazil, and from the states of Bahia, Minas Gerais, Espírito Santo and Rio de Janeiro (Fig. 1).

#### Included genera.

The family includes the following eight genera: *Bourreria*, *Cortesia*, *Ehretia*, *Halgania*, *Keraunea*, *Lepidocordia* Ducke, *Rochefortia* and *Tiquilia* Pers. (Luebert et al. 2016).

#### Notes.

Our morphological concept of the Ehretiaceae is little changed from that of Luebert et al. (2016). The characters that differ are included in bold in the description above. The first of these is that the four species of *Keraunea* are the first lianescent species included within the family (versus perennial herbs, shrubs, or trees). Lianas are elsewhere found in the Boraginales in the Cordiaceae (*Cordia* L.) and Heliotropiaceae (*Tournefortia* L.). Secondly, we have expanded the concept of the Ehretiaceae to include species with a few-flowered corymb inflorescence structure and where the flower and later fruit are inserted at the centre of an accrescent bracteole. To our knowledge, these characters are unique among not just the Ehretiaceae but the Boraginales, whose members are known for their characteristic scorpioid cymose inflorescences. We suggest the few-flowered inflorescence of *Keraunea* is the result of secondary reduction rather than a retained ancestral form. Within the Boraginales, reductions to few-flowered inflorescences are present elsewhere in the Boraginaceae, Codonaceae and Wellstediaceae (Luebert et al. 2016).

### ﻿ Keraunea

Taxon classificationPlantaeBoraginalesEhretiaceae

Cheek & Simão-Bianchini, Nord. J. Bot. 31: 454. 2013.

D044E34F-4348-5F7E-B9A4-911B8AF62E3E

#### Type species.

*Kerauneabrasiliensis* Cheek & Simão-Bianchini.

#### Description.

Scandent shrubs or lianas. Stems woody, cylindrical, hollow, lacking lenticels. Stipules lacking. Leaves on the main stems alternate, simple, petiolate; margins entire; venation pinnate, camptodromous or brochododromous. Side shoots with 2–4 aborted leaves, then 2–6 progressively larger leaves along the shoot. Inflorescences terminal on side shoots, determinate, corymbose, with 3–6 flowers; bracteoles one per flower or rarely lacking, leaf-like, inserted halfway along the pedicel. Flowers 5-merous, cosexual. Calyx campanulate, fused at the base, alternating with the petals; aestivation imbricate. Corolla with the tube campanulate, fused at the base; aestivation imbricate. Stamens epipetalous, inserted at the base of the corolla tube, alternating with the petals; filaments free; anthers basifixed, introrse, dehiscing via lateral slits; connectives extended. Nectary disk present, at base of the ovary. Ovary superior, subglobose, 2-locular, the locules biovulate, each with one functional and one aborted ovule. Style single, with two apical stigmas. Fruit a dry, indehiscent drupe, usually inserted in the centre of the persistent and accrescent bracteole; calyx persistent; corolla caducous; style persistent.

#### Distribution.

**Figure 2.**
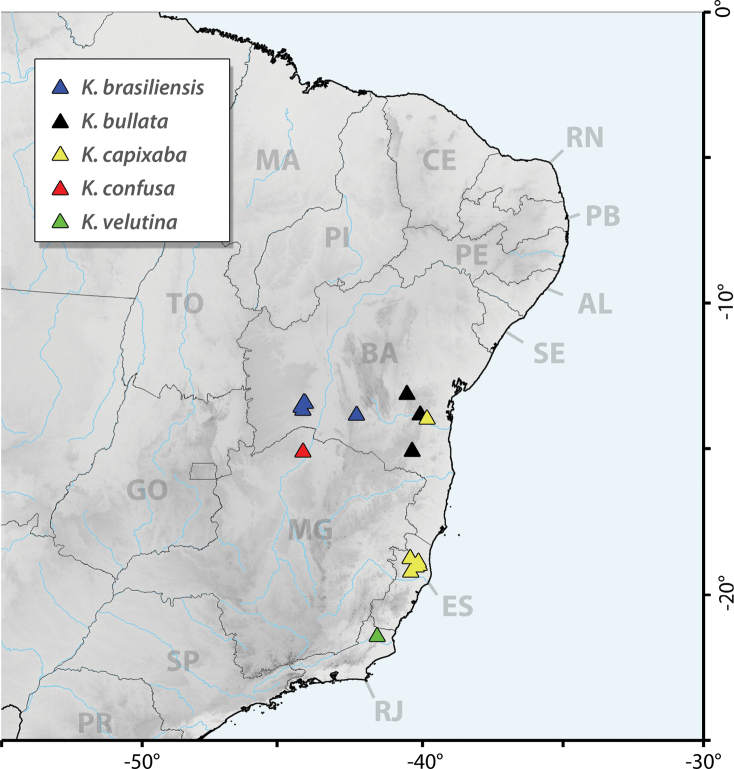
Distribution of species of the genus *Keraunea*. Shading denotes elevation; two letter codes represent the standardised two letter codes for the states of Brazil. Species are coloured as follows: **blue**, *K.brasiliensis* Cheek & Simão-Bianchini; **black**, *K.bullata* Moonlight & D.B.O.S.Cardoso; **yellow**, *K.capixaba* Lombardi; **red**, *K.confusa* Moonlight & D.B.O.S.Cardoso; **green**, *K.velutina* Moonlight & D.B.O.S.Cardoso.

*Keraunea* is endemic to Brazil and thus far known from the states of Bahia (3 spp.), Espírito Santo (1 sp.), Minas Gerais (1 sp.) and Rio de Janeiro (1 sp.). Each of the species is restricted to a single state except for *K.capixaba*, which is found in both Bahia and Espírito Santo (Fig. 2).

#### Habitat.

Two species are known from seasonally dry forest within the Caatinga domain (*K.brasiliensis* and *K.confusa*); two from humid forests within the Mata Atlântica domain (*K.capixaba* and *K.velutina*); and a fifth from transitional, semi-deciduous forests between the Caatinga and Mata Atlântica domains (*K.bullata*). The genus has a marked preference for rock outcrops or forest on rocky soils. Both *K.brasiliensis* and *K.confusa* are found mostly on karstic outcrops, while *K.capixaba* and *K.velutina* have both been collected at the base of granitic inselbergs.

#### Etymology.

The epithet derives from the Greek, keraunos, or lightning bolt. This was intended to signify the unexpected but now disproven appearance of a neuropeltoid genus of the Convolvulaceae in the Americas.

#### Notes.

Cheek and Simão-Bianchini (2013) treated the flower and fruit of *Keraunea* as being inserted at the centre of a large, leaf-like bract. This ‘bract’ is inserted halfway up and adnate to the pedicel, so we instead follow Lombardi (2014) and treat it as a bracteole rather than a bract.

#### Identification notes.

*Keraunea* species are distinctive in being semi-scandent shrubs or lianas with simple, alternate, exstipulate leaves and inflorescences terminal on foliose side shoots. The flowers and fruits are highly unusual in being inserted at and appearing to arise from the centre of an accrescent bracteole.

Several specimens of *Keraunea* were erroneously identified as *Bougainvillea* Comm. ex Juss. (Nyctaginaceae), which is understandable, as both genera have exstipulate leaves, a semi-lianescent habit, and flowers and fruits associated with showy bracts or bracteoles. *Keraunea* spp. are however never spinescent, and the flowers and fruits are inserted directly onto the bract, whereas in *Bougainvillea* several flowers or fruits are clustered on a pedunculate inflorescence surrounded by two or more bracts.

### ﻿Key to the species of *Keraunea*

**Table:** 

1	Leaf base rounded, truncate, or subcordate; leaf venation brochidodromous	**2**
–	Leaf base attenuate, acuminate, or obtuse; leaf venation camptododromous	**3**
2	Tertiary veins plane with the lower leaf surface, not raised; leaf blades to 18 × 12 cm, rugose	***Kerauneaconfusa***
–	Tertiary veins raised from the lower leaf surface; leaf blades to 10.5 × 7.5 cm, bullate	***Kerauneabullata***
3	Leaves on side shoots elliptic with attenuate apices; leaf blades conspicuously velutinous below	***Kerauneavelutina***
–	Leaves on side shoots ovate to broadly ovate with acute to obtuse apices; leaf blades glabrescent to hirtellous below	**4**
4	Main stem frequently branching, rugose; stems and leaves hirtellous; leaves membranous to papyraceous, dull green above; secondary veins raised abaxially	***Kerauneabrasiliensis***
–	Main stem rarely branching, smooth; stems and leaves glabrous; leaves chartaceous, glossy green above; secondary veins not raised abaxially	***Kerauneacapixaba***

### ﻿1. Keraunea brasiliensis

Taxon classificationPlantaeBoraginalesEhretiaceae

Cheek & Simão-Bianchini, Nord. J. Bot. 31: 454. 2013.

63FD551B-251F-5ABD-AB19-DA6D0314B7E1

Fig. 3

#### Type.

**Brazil. Bahia**: Mun. Caetité, caminho da Fazenda Boa Vista para Urânio, 13°59'35"S, 42°12'27"W, 560 m, 8 Feb 1997, *L. Passos*, *M.L. Guedes*, *B. Stannard & E. Saar 5263* (holotype: SPF; isotypes ALCB, CEPEC [CEPEC00077827], HRCB [acc. #38156], HUEFS [HUEFS000058973], K [K000979156]).

#### Description.

Scandent shrub or liana, to 7 m tall. Stems cylindrical, hollow, 3–9 mm in diameter, frequently branching, sericeous-pubescent with silky hairs; internodes 0.7–11 cm long; side shoots 4–9 cm long, hirtellous-pubescent with minute, rigid hairs. Leaves of main stem with the blades 5.0–6.5 × 3.1–3.6 cm, ovate, hirtellous-pubescent with minute, rigid hairs, the venation pinnate, camptodromous, with 4–6 secondary veins; the base acute, the margins entire, ciliate, the apex obtuse; petioles of leaves on main stem ca. 8 mm long, distinctly curved to 90° ⅓ of the way along their length. Side shoots with 3–5 leaves, these progressively larger along the shoot; blades 5.1–7.2 × 2.3–3.9 cm, ovate, hirtellous-pubescent, the venation as on leaves of the main stem, the base acute, the margins entire, ciliate, the apex obtuse; petioles 5.7–6.1 mm long, straight, hirtellous-pubescent like the leaves. Inflorescence corymbose, with 2–4 flowers; free portion of the peduncle ca. 3.5 mm long, 2.5 cm long, the remainder adnate to the bracteole, hirtellous-pubescent; bracteole 5.1–8.1 × 2.4–3.8 cm, inserted ca. ¼ of the way along the pedicel, ovate, hirtellous-pubescent, the venation as the leaves, the base acute, the margins entire, ciliate, the apex obtuse. Flowers incompletely known, 5-merous. Calyx with the tube campanulate, ca. 3.5 mm long, the lobes ca. 12 × 1.5 mm, lanceolate, velutinous-pubescent. Corolla unknown; androecium and gynoecium unknown. Fruit inserted on the accrescent bracteole; calyx persistent, expanding to 14 mm long.

**Figure 3.**
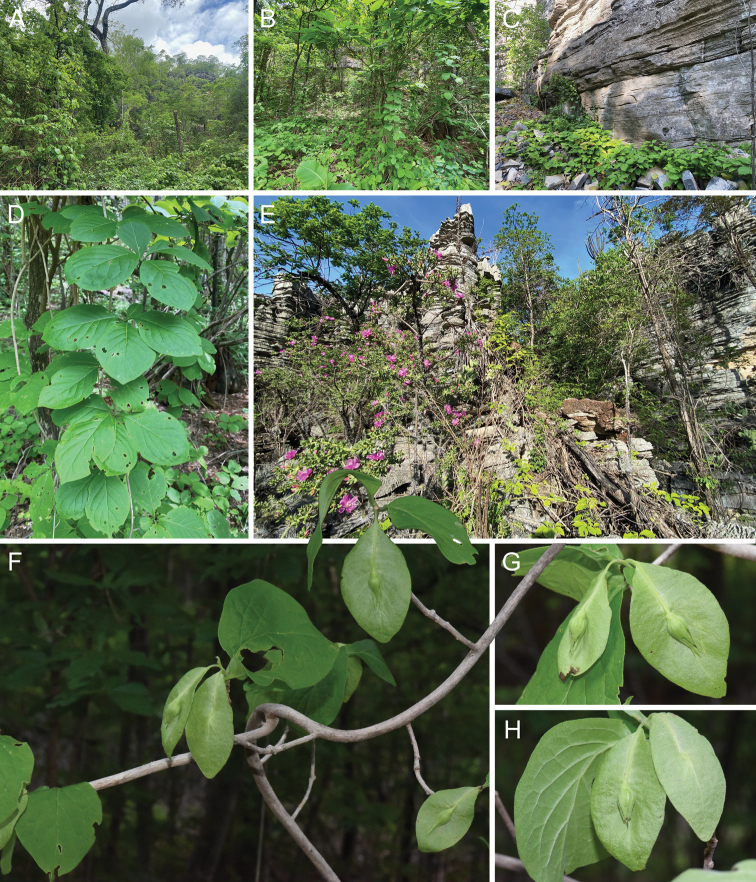
Habitat and morphology of *Kerauneabrasiliensis* Cheek & Simão-Bianchini **A–C** karstic Caatinga in southwestern Bahia **D** stem and leafy side shoots **E** habit **F** main shoot and side shoots with fruits **G–H** close up of developing fruits and accrescent bracteole. All photos by Domingos Cardoso.

#### Distribution.

*Kerauneabrasiliensis* is endemic to Brazil and is known only from the state of Bahia (Coribe, São Félix do Coribe and Caetité municipalities) (Fig. 2).

#### Habitat.

*Kerauneabrasiliensis* has primarily been collected scrambling over exposed rocks and vegetation in karst formations supporting Caatinga seasonally dry tropical forest vegetation. The two most easterly collections of the species (*Pereira-Silva et al. 9111*, *Passos et al. 5263*) were collected in Caatinga vegetation but far from any known karst outcrops.

#### Conservation status.

*Kerauneabrasiliensis* was provisionally assessed by Cheek and Simão-Bianchini (2013) as Endangered (EN B2 a b iii) under IUCN Criteria (IUCN 2019). We alter this assessment as it covered specimens now known to be of two different species. Our narrower concept includes plants from four localities and has an EOO of 4,550 km^2^. None of the known localities are in protected areas. Accordingly, we still assess *K.brasiliensis* as EN (EN B2 a b iii) under IUCN criteria (IUCN 2019).

#### Etymology.

Named for the country of Brazil.

#### Notes.

The protologue of *K.brasiliensis* cited individuals we now recognise as two different species and both paratypes collected in Minas Gerais fall under our circumscription of *K.confusa*. The authors of *K.brasiliensis* did, however, note the unusual intraspecific morphological variation within the species and speculated that the material collected in Bahia and Minas Gerais may have represented two different species. The original illustration in the protologue (Cheek and Simão-Bianchini 2013) included individuals of both of the species we recognise here so only the subfigures (B, H–I) represent *K.brasiliensis*. Muñoz-Rodríguez et al. (2022) also published sequence data for *K.brasiliensis* and *K.confusa* and a member of the family Malpighiaceae under the name *K.brasiliensis*.

Our emended description of *K.brasiliensis* reflects our much narrower circumscription of this species. The specimens we cite represent a morphologically homogenous group of specimens all collected at a similar phenological state, i.e. with fruits but lacking flowers. As such, our description lacks some floral traits. The floral description of *K.brasiliensis* provided by Cheek and Simão-Bianchini (2013) was made using a dissected bud from *Lombardi & Salino 1819* (K), which we now consider as an isotype of *K.confusa* (see below).

#### Identification notes.

Within its range, *K.brasiliensis* is most likely to be confused with *K.confusa*, but the two species can be distinguished with ease based entirely upon vegetative characters. The leaves of *K.confusa* are larger (to 18 × 12 cm versus to 7.5 × 4 cm) with brochidodromous venation and a rugose texture (versus camptodromous venation and smooth texture in *K.brasiliensis*). *Kerauneabrasiliensis* is most similar to *K.capixaba* (though this species is also similar to *K.velutina*), from which it can be distinguished by its rugose stems (versus smooth) and membranous, dull green leaves (versus chartaceous, glossy green) with indistinct secondary veins (versus raised above the lower leaf surface).

#### Additional specimens examined.

**Brazil. Bahia**: Mun. Coribe, BA-172 sentido São Félix do Coribe, entrada a direita em Colônia do Formoso por estrada de terra até afloramento de calcário a ca. 2.5 km de Ponta d’Água, 13°41'28"S, 44°15'33"W, 4 Dec. 2022, 4 Dec 2022, *D. Cardoso et al. 4902* (ALCB, RB, TCD), *D. Cardoso et al. 4904* (ALCB, HUEFS, RB, TCD), *D. Cardoso et al. 4906* (ALCB, HUEFS, RB, TCD; Mun. São Félix do Coribe, BA-172 sentido Coribe, afloramento de calcário do lado esquerdo, 13°26'6"S, 44°13'5"W, 4 Dec. 2022, *D. Cardoso et al. 4909* (ALCB, HUEFS, RB, TCD), *D. Cardoso et al. 4910* (ALCB, HUEFS, RB, TCD), *D. Cardoso et al. 4912* (ALCB, HUEFS, RB, TCD); Mun. Caetité, Distrito de Maniaçu, Estrada para São Timóteo, km 6, 13°51'18"S, 42°20'45"W, 870 m alt., 22 May 2004, *G. Pereira-Silva et al. 9111* (HUEFS [HUFES000117294]); Mun. Santa Maria da Vitória, c. 7.7 km S de Santa Maria da Vitória na Estrada para Lagoinha, extremidade septentrional da Serra do Ramalho, 13°27'0"S, 44°10'16"W, 13 Feb 2000, *L.P. de Queiroz et al. 5972* (CEPEC [CEPEC00113512]; HUEFS [HUEFS000110252]); Mun. Coribe, c. 5 km S em estrada de terra que cruza pequeno ramal que sai a 5.1 km E de Ponta d’Água, a 24.4 km S de São Félix do Coribe na estrada para Coribe, 13°35'10"S, 44°19'12"W, 14 Apr. 2007, *L.P. de Queiroz et al. 12707* (CEN [CEN00113310], HUEFS [HUEFS000117258]).

### ﻿2. Keraunea bullata

Taxon classificationPlantaeBoraginalesEhretiaceae

Moonlight & D.B.O.S.Cardoso sp. nov.

03C5023D-3582-5348-8BAC-7D5F32CE2A13

urn:lsid:ipni.org:names:77314429-1

Fig. 4

#### Diagnosis.

Most similar to *K.confusa* with relatively broad leaves with brochidodromous venation but differing from it in smaller leaves (reaching 10 × 7.4 cm versus 18 × 12 cm) with a bullate texture (versus rugose) and by its tertiary veins, which are raised from the lower leaf surface (versus not raised).

#### Type.

**Brazil. Bahia**: Mun. Jequié, Bairro Suíça, 13°51'S, 40°5'W, *M.L. Guedes*, *D.M. Loureiro & D.L. Santana 9176* (holotype: ALCB [acc. #55929, ALCB075866, 3 sheets]).

#### Description.

Scandent shrub or liana, to 2 m. Stems cylindrical, hollow, 2–5 mm in diameter, frequently branching, glabrescent to sparsely pubescent; internodes 2.9–8.2 cm long; side shoots 3.0–4.5 cm long, sericeous. Leaves of main stem unknown. Side shoots with 3–4 leaves, these progressively larger along the shoot; blades 4.2–10.5 × 2.4–7.4 cm, ovate to broadly ovate, velutinous-pubescent, the venation pinnate, brochidodromous, with 4–6 secondary veins; the base rounded to truncate, the margins entire, ciliate, the apex obtuse; petioles 8–19 mm long, straight, velutinous-pubescent. Inflorescence corymbose, with 2–6 flowers; free portion of the peduncle 4–6 mm long, 1.8–2.1 cm long, the remainder adnate to the bracteole, sericeous-pubescent; bracteoles 5.0–5.7 × 2.7–3.1 cm, inserted ca. ¼ of the way along the pedicel or rarely lacking, ovate, velutinous-pubescent, the venation as the leaves, the base short-rounded, the margins entire, ciliate, the apex attenuate. Flowers incompletely known, 5-merous. Calyx with the tube campanulate, ca. 1.5 mm long, the lobes ca. 1.5 × 10–12 mm, ensiform, sericeous-pubescent. Corolla unknown; androecium and gynoecium unknown. Fruit inserted on the accrescent bracteole; calyx persistent, expanding to 12 mm long.

**Figure 4.**
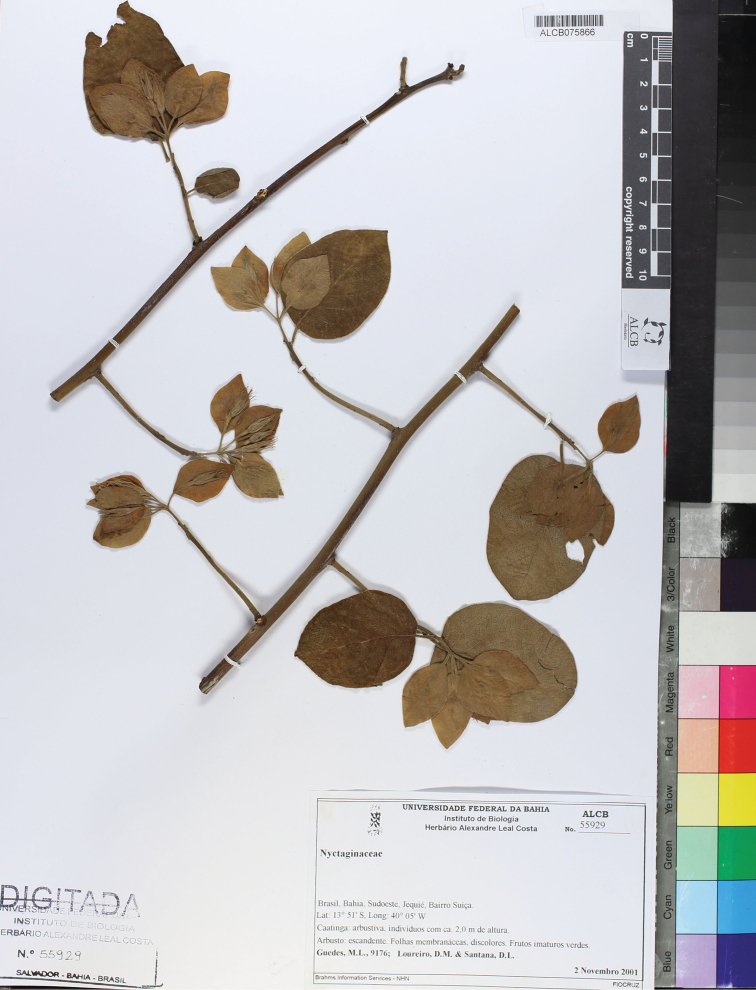
Holotype of *Kerauneabullata* Moonlight & D.B.O.S.Cardoso, *M.L. Guedes*, *D.M. Loureiro & D.L. Santana 9176* (ALCB [ALCB075855, acc. #55929, sheet 1]).

#### Distribution.

Endemic to the state of Bahia (Fig. 2).

#### Habitat.

*Kerauneabullata* is a species of forests in the transition from humid forests in the coastal Mata Atlântica phytogeographic domain and seasonally dry forests in the inland Caatinga domain. This forest is described on labels as “Caatinga arborea” (*Guedes et al. 9176*) and (*Mori et al. 11534*) as growing on rocky soils. This area has a metamorphic bedrock (often called crystalline in the literature, de Queiroz et al. 2017) and is quite edaphically distinct from the karstic outcrops that are home to *K.brasiliensis* and *K.confusa*.

#### Conservation status.

*Kerauneabullata* is known from only three localities with a combined EOO of ca. 6,300 km^2^. We know of no specific conservation threats to this species so provisionally assess it as Vulnerable (VU D2) under IUCN Red List criteria (IUCN 2019).

#### Etymology.

Named for the bullate texture of the leaves and bracteoles.

#### Identification notes.

*Kerauneabullata* is highly distinct within its range. Despite also occurring in the same Mata Atlântica phytogeographic domain as *K.capixaba*, their habitat does not overlap. *Kerauneabullata* can be morphologically distinguished by its leaf venation (brochidodromous versus camptodromous) and texture (bullate and dull green versus smooth and glossy green).

#### Additional specimens examined.

**Brazil. Bahia**: Mun. Itambé, rod. BA-265, trecho Itapatinga/Caatiba, Faz. Serra Verde a 17 km da Rod. BR-415, [15°6'S, 40°22'W], 14 Mar. 1979, *S.A. Mori et al. 11534* (CEPEC [CEPEC00016207], NY [03147645], RB [00725192]). Mun. Juraci, Vicinity of Machado Portela [13°10'S, 40°46'W], 19–23 Jun 1915, *J.N. Rose & P.G. Russell 19979* (US [01341072, acc. # 762304).

### ﻿3. Keraunea capixaba

Taxon classificationPlantaeBoraginalesEhretiaceae

Lombardi, Phytotaxa 181: 54. 2014.

38DDCC77-E08B-56E9-8AD3-83390FFB49FC

Fig. 5

#### Type.

**Brazil. Espírito Santo**: Mun. Jaguaré, perto da Comunidade São Jorge de Paduá, sentido para Fátima, 18°54'29.0"S, 40°8'44.9"W, 26 Sep. 2013, *G.S. Siqueira 891* (holotype: CVRD [acc. # 14565]; isotypes HRCB [acc. # 76196], SP [SP003725]).

#### Description.

Scandent shrub or liana, to ca. 4 m. Stems cylindrical, hollow, 2–4 mm in diameter, glabrescent, rarely branching, internodes 2.4–11.5 cm long; side shoots 1.3–5.5 cm long, glabrescent. Leaves of main stem with the blades 7.0–13.5 × 2.1–4.5 cm, elliptic, glabrescent, the venation pinnate, camptodromous, with 4–6 secondary veins; the base cuneate, the margins entire, ciliate, the apex attenuate; petioles 5–6.5 mm long, straight. Side shoots with 4–6 leaves, these progressively larger along the shoot; blades 1–9.5 × 0.5–6.5 cm, ovate, rarely obovate or broadly ovate, glabrescent, the venation as on leaves of the main stem, the base cuneate to rounded, the margins entire, rarely crenulate toward the apex (*Siqueira 891*), ciliate, the apex acute to rounded; petioles 5.5–12 mm long, straight, glabrescent. Inflorescence corymbose, with 2–4 flowers; free portion of the pedicel, 8–14 mm long, 1.8–2.6 cm long, the remainder adnate to the bracteole, sericeous-pubescent with silky hairs; bracteole 3.3–3.5 × 1.9–2.4 cm, inserted ca. ½ of the way along the pedicel or rarely lacking, ovate, glabrescent, the venation as the leaves, the base short acute, margin entire, not ciliate, the apex acute. Flowers 5-merous. Calyx with the tube campanulate, ca. 3.5 mm long, the lobes 6.5–18 × 1.5–2.5 mm, ensiform, glabrescent to minutely pubescent. Corolla with the tube campanulate, ca. 6.5 mm long, the lobes 19–26 × 7.5–11.5 mm, elliptic to oblong, glabrous. Stamens epipetalous, inserted at the base of the corolla tube, the filaments 2–3 mm long, the anthers ca. 9 mm long, connective extending to 3.2 mm. Ovary subglobose, 2-locular, the locules biovulate; style single, conduplicate, unbranched; stigmas 2, truncate. Fruit inserted on the accrescent bracteole; calyx persistent, expanding to 18 mm long.

**Figure 5.**
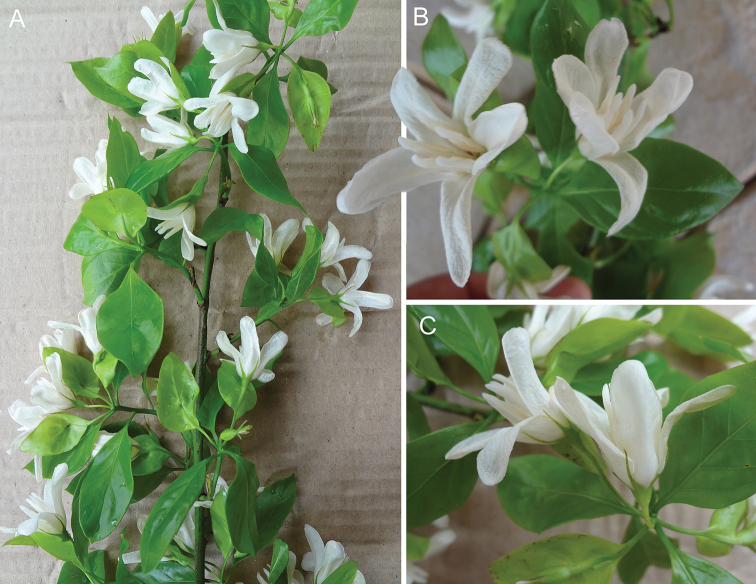
Morphology of *Kerauneacapixaba* Lombardi **A** habit **B** inflorescence, front view **C** inflorescence, side view. For photograph of fruit with accrescent bracteole see Lombardi (2014). All photographs by Geovane Siqueira.

#### Distribution.

Endemic to Espírito Santo state and known from the municipalities of Jaguaré, Sooretama and Nova Venécia (Fig. 2).

#### Habitat.

Collections of *Kerauneacapixaba* have primarily been made in “mata tabuleiro” or flat, semi-deciduous forests found within the coast of the Mata Atlântica domain of Brazil. A more recent collection (*Gurtler & Dutra 371*) was made growing over rocks in forest understory at the base of a granitic outcrop. Specimen labels describe the species as scandent or lianescent.

#### Conservation status.

*Kerauneacapixaba* was provisionally assessed by Lombardi (2014) as Endangered because it was at that time known from four collections made within disturbed forest patches adjacent to the Sooterama Biological Reserve. We add five collections to the known distribution, including one collected within the Sooterama Biological Reserve (*Covre s.n.*), two collected at the base of inselbergs > 30 km from the reserve (*Gurtler & Dutra 371*, *Demuner et al. 3799*), and a gathering made a remarkable 500 km to the north near Ipauí, Bahia. The species is now known from six localities with a collective Extent of Occurrence (EOO) of ca. 11,000 km^2^. The species is known from < 10 localities and has a EOO of < 20,000 km^2^, so we assess *K.capixaba* as Vulnerable (VU B1 a b iii) under IUCN criteria (IUCN 2019).

#### Etymology.

The epithet is an indigenous term referring to people or objects from Espírito Santo state.

#### Identification notes.

*Kerauneacapixaba* is most morphologically and ecologically similar to the newly described *K.velutina*, the two species of the genus that are associated with more humid settings in the Mata Atlântica phytogeographic region, yet they were never found growing sympatrically. *Kerauneacapixaba* differs in lacking an indumentum on the stems, side shoots and lower leaf surface (versus a velutinous-pubescent in *K.velutina*), and its ovate leaves on the side shoots (versus elliptic to narrowly-lanceolate). Its range is close to that of *K.confusa*, from which it is readily distinguished by its camptodromous venation (versus brochidodromous).

#### Additional specimens examined.

**Brazil. Bahia**: Rod Ipiau-Ibirataia, [14°6'S, 39°41'W], 13 Nov. 1971, *T.S. Santos 2139* (HUEFS). **Espírito Santo**: Mun. Sooretama, Reserva Biológica de Sooretama, [19°1'S, 40°7'W], 19 May 2015, *C. Covre s.n.* (SAMES [SAMES03696]); Mun. Rio Bananal, Alto Bananal, 19°14'56"S, 40°24'59"W, 300–600 m alt., 25 Apr. 2007, *V. Demuner et al. 3799* (BHCB [BHCB017211]); Mun. Jaguaré, Perto da Comunidade São Jorge de Paduá, 25 Sep. 2013, *D.A. Folli 7117* (CVRD [acc. #14563]; HRCB [acc. #16194], NY [02687787], RB [00895205]); Mun. Sooretama, Barro Roxo a Córrego Rodrigues, 19°4'35"S, 40°13'25"W, 156 m alt., 8 Oct. 2014, *D.A. Folli 7273* (CVRD [acc. #15122]; RB [01103439]); Mun. Nova Venécia, fazenda Santa Rita, ao pé da pedra da torre (P3), 18°47'7"S, 40°26'29"W, 2 Feb. 2018, *J. Gurtler & S.C. Dutra 371* (VIES [VIES036853]); Mun. Sooretama, Rodovia ES 358, distrito de Bom Jardim, 19°2'20.31"S, 40°14'48.23"W, 1 Sep. 2012, *A. Moreira de Assis & J. Freitas 3340* (HRCB [acc. #76193], MBML [2 sheets: MBML00016754, MBML00016755]); Mun. Jaguaré, perto da Comunidade São Jorge de Paduá, 18°54'29.0"S, 40°8'44.9"W, 6 Oct. 2013, *G.S. Siqueira 893* (CVRD [acc. #14570], HRCB [acc. #76199], K [K001275507], MBML [MBML00016491], NY [02687786], RB [00895202])

### ﻿4. Keraunea confusa

Taxon classificationPlantaeBoraginalesEhretiaceae

Moonlight & D.B.O.S.Cardoso sp. nov.

2FAB84E9-474A-56EB-B7FF-B3B747DE60C7

urn:lsid:ipni.org:names:77314430-1

Fig. 6

#### Diagnosis.

Most similar to *K.brasiliensis* but differing in its larger leaves (to 18 × 12 cm versus to 7.5 × 4 cm) with brochidodromous (versus camptodromous) leaf venation and a rugose (versus flat) leaf texture and by its leaves and stems with a sericeous (versus hirtellous) indumentum.

#### Type.

**Brazil. Bahia**: Mun. Januária, distrito de Fabião, junto ao Abrigo do Malhador, 15°7.85'S, 44°15.17'W, 25 May 1997, *J.A. Lombardi & A. Salino 1819* (holotype: BHCB [BHCB017209]; isotypes: HRCB [acc. #26438]; K [K001395055], UEC [UEC117783]).

#### Description.

Scandent shrub or liana, to 4 m tall. Stems cylindrical, hollow, 2–5 mm in diameter, rarely branching, sericeous-pubescent with silky hairs; internodes 3.8–16.5 cm long; side shoots 2.6–3.5 cm long, sericeous-pubescent. Leaves of the main stem with the blades 9.5–17 × 5.6–13.5 cm, ovate, velutinous-pubescent, the venation pinnate, brochidodromous, with 5–7 secondary veins, the base truncate to subcordate, the margins entire, ciliate, the apex attenuate to acute; petioles of leaves on main stem 10–16 mm long, distinctly curved to 90° ¼ of the way along their length. Side shoots with 2–5 leaves, these progressively larger along the shoot; blades 2.1–18 × 3.0–12 cm, velutinous-pubescent, the venation as on leaves of the main stems, the base rounded to truncate, the margins entire, ciliate, the apex obtuse; petioles 3–25 mm long, sericeous-pubescent. Inflorescence corymbose, with 2–4 flowers; free portion of the peduncle 5–13 mm long, 2.2–2.8 cm long, the remainder adnate to the bracteole, sericeous-pubescent; bracteole 4.9–5.6 × 2.7–5.1 cm, inserted ca. ⅓ of the way along the pedicel or rarely lacking, ovate, sericeous-pubescent, the venation as the leaves, the base short cuneate, the margins entire, ciliate, the apex attenuate. Flowers incompletely known, 5-merous. Calyx with the tube campanulate, ca. 1.5 mm long, the lobes ca. 7–9 × 1.5 mm, ensiform, sericeous-pubescent. Corolla unknown; androecium and gynoecium unknown. Fruit inserted on the accrescent bracteole; calyx persistent, expanding to 9 mm long.

**Figure 6.**
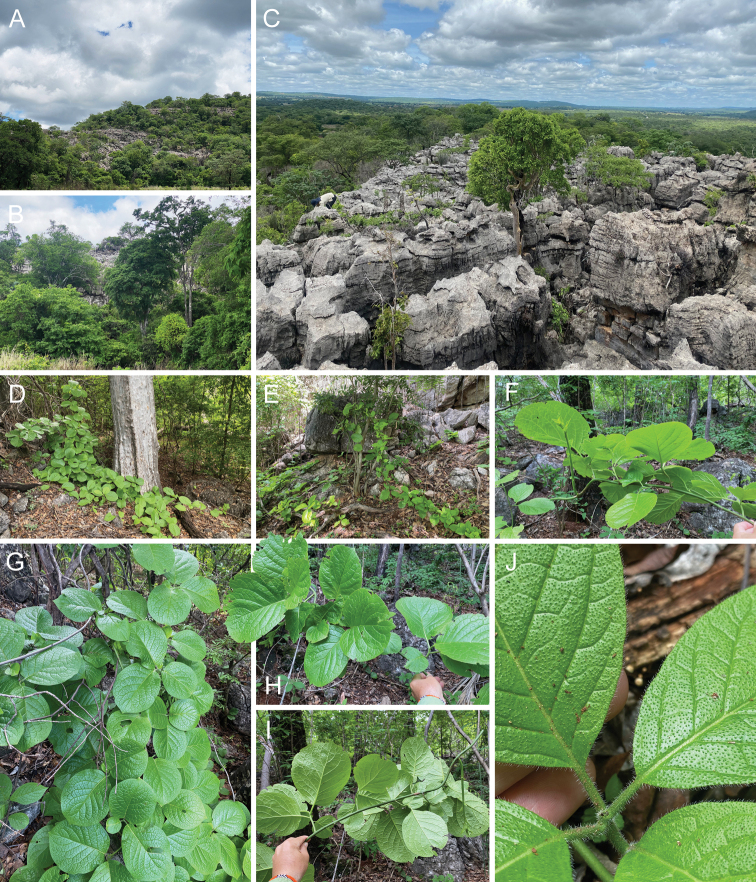
Habitat and morphology of *Kerauneaconfusa* Moonlight & D.B.O.S.Cardoso **A–C** karstic Caatinga seasonally dry tropical forest in northern Minas Gerais **D–G** habit **H–I** branching pattern **J** close up of side shoot. All photographs by Domingos Cardoso.

#### Distribution.

*Kerauneaconfusa* is endemic to Brazil and to the state of Minas Gerais (Fig. 2).

#### Habitat.

All collections of *K.confusa* are from karstic (limestone) areas, where plants have been collected growing over rocks and vegetation in the understory of Caatinga seasonally dry tropical forest vegetation.

#### Conservation status.

*Kerauneaconfusa* is known from two localities and has an AOO of ca. 12 km^2^. Two of the known collections (*Lombardi & Salino 1819*, *Lombardi 2107*) were made within Parque Nacional Cavernas do Peruaçu, which includes extensive karst limestone habitat suitable for the species. We know of no specific threats to this species but provide a preliminary assessment as Vulnerable (VU D2) because of the species’ few known localities (IUCN 2019).

#### Etymology.

The epithet refers to the confusion that has surrounded the taxonomic history of this species, which was both included within the original circumscription of the type species and the placement of the genus in the wrong family by Cheek and Simão-Bianchini (2013).

#### Identification notes.

*Kerauneaconfusa* is one of two species of the genus with brochidodromous venation, the other of which is *K.bullata*. It is distinguished from *K.bullata* by its generally bigger leaves measuring 9.5–17 cm long (versus 4.2–10.5 cm long), rugose (versus bullate) leaf texture and by the tertiary veins, which are plane to the lower leaf surface (versus raised).

#### Additional specimens examined

**(paratypes). Brazil. Minas Gerais**: Mun. Manga, BR-135, entre Montalvânia e Manga, afloramento de calcário do lado esquerdo a ca. 400 m da rodovia sentido Manga-Januária, 14°30'44.8"S, 44°10'31.8"W, 5 Dec. 2022, *D. Cardoso et al. 4916* (ALCB, HUEFS, TCD, RB), *D. Cardoso et al. 4918* (ALCB, HUEFS, TCD, RB); Mun. Januária, distrito de Fabião, junto ao Abrigo do Malhador, 15°7'16”–15°8'57"S, 44°15'20”–44°14'13"W, 26 Oct. 1997, *J.A. Lombardi 2107* (BHCB [BHCB017210], K [K000593363]).

### ﻿5. Keraunea velutina

Taxon classificationPlantaeBoraginalesEhretiaceae

Moonlight & D.B.O.S.Cardoso sp. nov.

D7C372AE-2461-516C-BBEC-D3DDCD808DAD

urn:lsid:ipni.org:names:77314431-1

Fig. 7

#### Diagnosis.

Most similar to *Kerauneacapixaba* but differing in its velutinous indumentum on the stems, side shoots and lower leaf surface (versus glabrescent); its strigose indumentum on the upper leaf surface (versus glabrescent); its elliptic to narrowly lanceolate leaves on the side shoots (versus ovate).

#### Type.

**Brazil. Rio de Janeiro**: Mun. Cardoso Moreira, Santíssimo, Fazenda Borges, [21°29'S, 41°37'W], 10 Sep. 2013, *J.G. Costa 257* (holotype: RB [00852871]).

#### Description.

Scandent shrub or liana, size unknown. Stems cylindrical, hollow, 2–3 mm in diameter, rarely branching, velutinous-pubescent; internodes 2–5.9 cm long; side shoots 2.2–4.7 cm long velutinous-pubescent. Leaves of the main stem with the blades 7.9–8.1 × 2.7–3.1 cm, lanceolate, velutinous-pubescent, the venation pinnate, camptodromous, with 6–8 secondary veins; the base obtuse, the margins entire, ciliate, the apex attenuate; petioles of leaves on main stem 10–11 mm long, distinctly curved to 90° ¼ of the way along their length. Side shoots with 6–8 leaves, these progressively larger along the shoot; blades 0.5–3.5 × 0.3–1.7 cm, elliptic, rarely or lanceolate or obovate, strigulose-pubescent above, velutinous below, the base cuneate to rounded, the margins entire, ciliate, the apex rounded to attenuate. Inflorescence corymbose, with 2–4 flowers; free portion of the peduncle 3–5 mm long; pedicel 0.5–1.5 cm long, the remainder adnate to the bracteole, sericeous-pubescent; bracteole 2–3.8 × 0.7–1.6 cm, inserted ca. ⅓ of the way along the pedicel or rarely lacking, ovate, velutinous- pubescent, the venation as the leaves, the base short-cuneate, margin entire, ciliate, the apex attenuate. Flowers 5-merous. Calyx with the tube campanulate, ca. 1.5 mm long, the lobes 0.7–1.4 × 6.7–12 mm, ensiform, velutinous-pubescent. Corolla with the tube campanulate, 5–5.9 mm long, the lobes 14 × 7 mm, elliptic, glabrous. Stamens epipetalous, inserted at the base of the corolla tube, filaments 3–4 mm long, anthers ca. 7 mm long, connective extending to 1.3 mm; ovary subglobose, 2-locular, the locules biovulate; style single, conduplicate, unbranched; stigmas 2, truncate. Fruit inserted on the accrescent bracteole; calyx persistent, expanding to 15 mm long.

**Figure 7.**
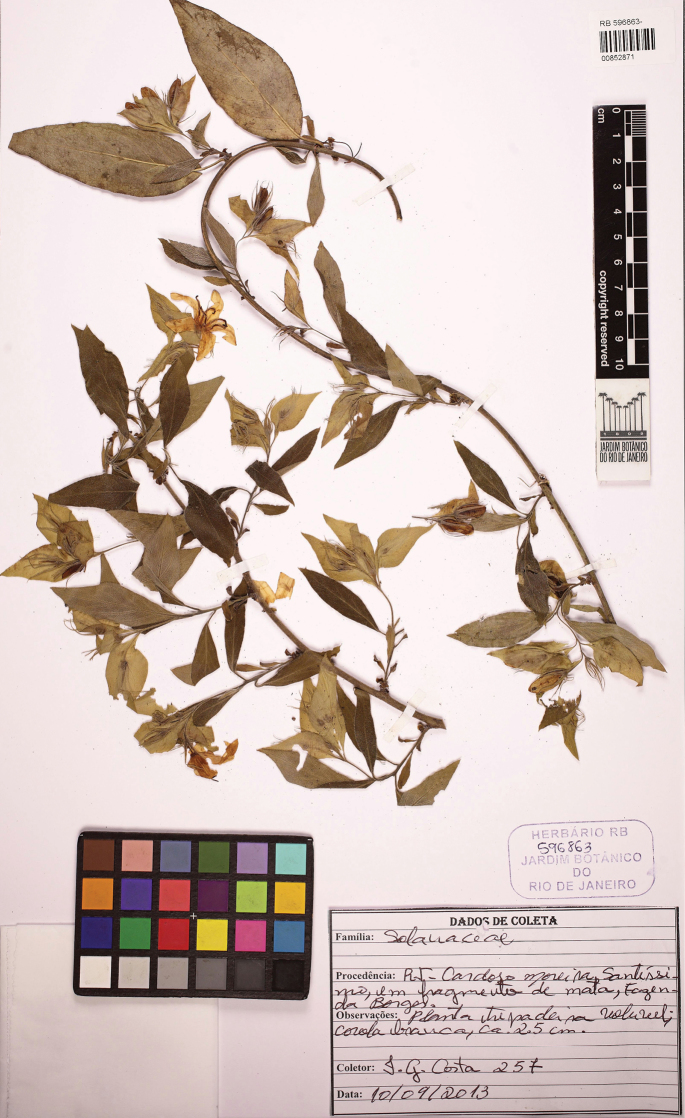
Holotype of *Kerauneavelutina* Moonlight & D.B.O.S.Cardoso, *J.G. Costa 257* (RB [00852871]).

#### Distribution.

*Kerauneavelutina* is endemic to Brazil and known only from the type from Cardoso Moreira municipality in the state of Rio de Janeiro (Fig. 2).

#### Habitat.

The single specimen known of *K.velutina* was collected within a forest fragment around isolated inselbergs. Cardoso Moreira municipality includes several granitic inselbergs and deforestation appears reduced around these compared to the surrounding land. We suspect that, like the similar *Kerauneacapixaba*, this species was collected scrambling over rocks at the base of an inselberg.

#### Conservation status.

*Kerauneavelutina* is known from a single specimen collected within a small forest fragment. Forest cover within Cardoso Moreira municipality declined from 5.2% in 1985 to 4.0% in 2012 before recovering to 4.6% by 2021 (Mapbiomas 2022). This picture is however complex, with Google Earth imagery showing that deforestation of primary forest is continuing in some areas, but forest is recovering in other areas (Google Earth Pro 2020). As such, due to single collection with imprecise locality information from a largely deforested municipality, we provisionally assess *K.velutina* as Critically Endangered (CR B1 B2 a b ii) under IUCN criteria (IUCN 2019).

#### Etymology.

Named for the plant’s velvety indumentum.

#### Identification notes.

*Kerauneavelutina* is a distinct species easily distinguished from all others in the genus by the dense, velvety indumentum of the stems and lower leaf surfaces. It is also distinctive in its side shoots with more numerous leaves than other members of the genus (6–8 versus 2–5), which are almost all elliptic (versus variously ovate to broadly ovate).

## Supplementary Material

XML Treatment for Ehretiaceae

XML Treatment for Keraunea

XML Treatment for Keraunea brasiliensis

XML Treatment for Keraunea bullata

XML Treatment for Keraunea capixaba

XML Treatment for Keraunea confusa

XML Treatment for Keraunea velutina

## Appendix 1

**Index to collections**

Only first collectors are cited.

Cardoso, D. 4902, 4904, 4909, 4910, 4912 (brasiliensis); 4916, 4918 (confusa)

Costa, J.G. 257 (velutina)

Covre, C. s.n. (capixaba)

Demuner, V. 3799 (capixaba)

Folli, D.A. 7117, 7273 (capixaba)

Guedes, D.M. 9176 (bullata)

Gurtler, J. 371 (capixaba)

Lombardi, J.A. 1819, 2107 (confusa)

Moreíra de Assis, A. 3340 (capixaba)

Mori, S.A. 11534 (bullata)

Passos, L. 5263 (brasiliensis)

Pereira-Silva, A. 9111 (brasiliensis)

de Queiroz, L.P. 5972, 12707 (brasiliensis)

Rose, J.N. 19979 (bullata)

Santos, T.S. 2139 (capixaba)

Siqueira, G.S. 891, 893 (capixaba)
